# Supplementary material for: Co-Spray Drying of Paracetamol and Propyphenazone with Polymeric Binders for Enabling Compaction and Stability Improvement in a Combination Tablet
Source: Pharmaceutics. 2021 Aug 14;13(8):1259. doi: 10.3390/pharmaceutics13081259 (PMC8399363; doi:10.3390/pharmaceutics13081259)
Supplement: Supplementary file 1 [file pharmaceutics-13-01259-s001.zip › pharmaceutics-1317781-supplementary.pdf]

pharmaceutics

MDPI

Supplementary Material

Supplementary Material: Co-Spray Drying of Paracetamol and Propyphenazone with Polymeric Binders for Enabling Compaction and Stability Improvement in a Combination Tablet

Ioannis Partheniadis, Ioannis Nikolakakis, Constantinos K. Zacharis, Kyriakos Kachrimanis and Nizar Al-Zoubi

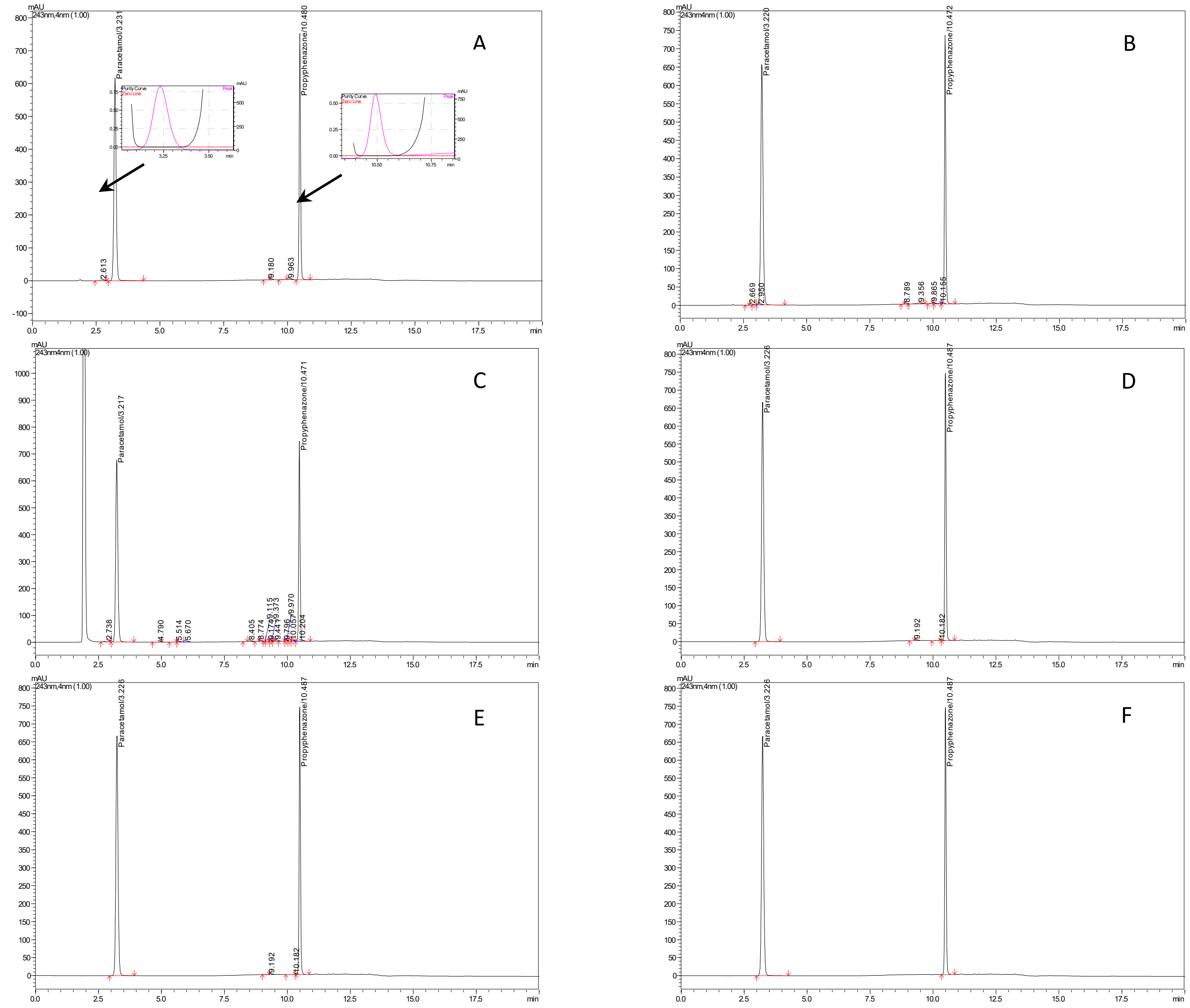

Figure S1. Chromatograms showing the specificity of the developed HPLC method for PCT (100 µg/mL), PRP (100 µg/mL) and their degradation products under **A**) acidic conditions (1M HCl), **B**) basic conditions (1 M NaOH), **C**) oxidative conditions (3 % *w/w* H_2O_2), **D**) hydrolytic conditions, **E**) photolytic conditions (sunlight exposure), **F**) thermal conditions (60 ^{o}C). The inserts present the peak purity curve for the respective analyte peak.
